# Phenotypical Differences at the Physiological and Clinical Level between Two Genetically Closely Related *Clavispora lusitaniae* Strains Isolated from Patients

**DOI:** 10.3390/jof10070460

**Published:** 2024-06-28

**Authors:** Debora Casagrande Pierantoni, Simone Giuliano, Angela Conti, Laura Corte, Jacopo Angelini, Gianluigi Cardinali, Carlo Tascini

**Affiliations:** 1 Department of Pharmaceutical Sciences, University of Perugia, 06121 Perugia, Italy; debora.casagrandepierantoni@unipg.it (D.C.P.); angela.conti@unipg.it (A.C.); laura.corte@unipg.it (L.C.); 2 Infectious Diseases Division, Department of Medicine (DMED), University of Udine and Azienda Sanitaria Universitaria Friuli Centrale, 33100 Udine, Italy; simone.giuliano@asufc.sanita.fvg.it (S.G.); jacopo.angelini@asufc.sanita.fvg.it (J.A.); carlo.tascini@uniud.it (C.T.); 3 CEMIN Excellence Research Centre, 06123 Perugia, Italy; 4 Clinical Pharmacology and Toxicology Institute, University Hospital Friuli Centrale ASUFC, 33100 Udine, Italy

**Keywords:** *Clavispora lusitaniae*, antifungal resistance, ITS, physiological variability

## Abstract

The occurrence of non-albicans species within the genus *Candida* poses a major challenge in the clinical setting. *Clavispora lusitaniae*, formerly known as *Candida lusitaniae*, has gained attention due to its potential multidrug resistance, particularly to amphotericin B (AmB). While intrinsic resistance to AmB is rare, secondary resistance may develop during treatment due to phenotypic rearrangement and the reorganization of the cell wall. Although there is evidence of genetic variability within *C. lusitaniae*, comprehensive genomic studies are lacking. This study examines the physiological differences within *Candida* species and focuses on the medical implications of this. Using two case reports, significant physiological and resistance differences between two strains of *C. lusitaniae* are demonstrated, highlighting the need for further research into genetic variability. While one strain showed higher resistance to antifungal drugs and slower growth compared to Strain 2, both strains showed minimal beta-D-glucan production, suggesting alternative pathogenic mechanisms. The study underlines the importance of understanding microbial adaptation and selection mechanisms, especially in the clinical setting, to effectively combat emerging drug resistance. Furthermore, research is needed to clarify the complex interplay between environmental causes, physiological traits, and the mechanisms of drug resistance in *C. lusitaniae*.

## 1. Introduction

The members of the pathogenic species of the genus *Candida* are usual commensal fungi from human microbiota and have been found in expectorated sputum, bronchoalveolar lavage fluid, the gastrointestinal tract, female genital tract, the urine of patients with indwelling Foley catheters and the skin [1,2]. Whereas in the past, *Candida albicans* was the dominant pathogen, other species of this group are now rising as emergent opportunistic or pathogenic agents and *C. albicans* represents half of the detected isolates, with *Candida* non-albicans species (NCAC) accounting for the other 50% [3]. Among NCAC, *Clavispora lusitaniae*, known as *Candida lusitaniae* [4] before the introduction of the “one fungus one name” criterion [5], has been recognized as an emerging potential for multi-drug resistance (hereinafter referred to as MDR, i.e., resistant to at least one antifungal agent and belonging to at least two antifungal drug classes) [6] with the propensity to cause invasive fungal infections in the immunocompromised host [7]. Although *C. lusitaniae* has been considered intrinsically resistant or tolerant to amphotericin B (AmB), it appears that AmB resistance is quite rare among incident candidemia isolates of *C. lusitaniae*, but secondary resistance to the drug might emerge during the course of treatment [8,9]. Resistance to AmB in *C. lusitaniae* might be associated with phenotypic switching [10]. Also, cell wall reorganization could induce resistance by decreasing the total amount of ergosterol or by substituting polyene-binding sterols with others displaying weaker affinity or reorientation or the masking of existing ergosterol molecules, which are all mechanisms predicted to make binding with polyenes less favorable thermodynamically and in terms of steric hindrance [11,12]. The first report that documented the emergence of a polyene-resistant strain initially identified as *Candida tropicalis* and later reidentified as *Candida lusitaniae* was in March 1979 [13]. The patient had undergone a bone marrow transplant, and the isolate developed resistance to AmB during antifungal therapy [13]. In May 1979, a second report described a candidemia caused by *C. lusitaniae* in a patient with acute myelogenous leukemia [14]. Notably, the isolate demonstrated the ability to acquire resistance to AmB during systemic treatment with AmB [14]. *C. lusitaniae* is a yeast species with strong variability of the LSU locus (26S), which is a fact that could be explained by a species split into two allopatric populations that finds confirmation in the Mycobank ITS-based tree showing two major clades [15]. The type strains CBS 4413 (*h^−^*) and CBS 6936 (*h^+^*) are placed in the two clades and were used in this work to identify the strains studied.

The presence of supposed genetic variability, as estimated with these two taxonomic markers, has not yet been analyzed by appropriate genomic studies to our knowledge. This work is based on the observations reported below in the two case reports and is based on the hypothesis that among members of the same species, significant physiological differences with relevant effects from the medical viewpoint can occur. 

## 2. Materials and Methods

### 2.1. Antifungal Susceptibility Testing and Strain Identification

We investigated the susceptibility of *Candida* strains isolated from the two patients to various antifungal drugs, including isavuconazole, fluconazole, and liposomal amphotericin B. Susceptibility testing was performed using the Card Vitek^®^ 2 AST and Etest with RPMI-2% glucose medium following the instructions provided by Biomérieux S.p.A., Florence, Italy. In addition, the susceptibility of planktonic cells to antifungals was determined according to the European Committee of Antimicrobial Susceptibility Testing (EUCAST) method for the determination of broth microdilution minimum inhibitory concentrations (MICs) of antifungal agents for yeasts [16], using the RPMI-1640 medium with the addition of 2% glucose. The MIC was assessed by examining the presence of a deposit or turbidity in the wells, following the EUCAST method. To ensure accuracy, the resazurin staining method was also used for additional confirmation [17]. To identify the strains, we employed MALDI-TOF MS (Bruker, Ettlingen, Germany) for identification. Before sample preparation, all yeasts were grown on CHROMagar at 37 °C for 2 days to exclude contamination by the main *Candida* species (i.e., *Candida albicans*). A colony was selected, fixed with 70% ethanol, and subjected to the addition of acetonitrile and 70% formic acid after ethanol evaporation. Then, 1 μL of the resulting supernatant was deposited on a suitable support. The dried spots were covered with Matrix HCCA (α-Cyano-4-hydroxycinnamic acid) from Bruker, Germany, and allowed to dry at room temperature. Finally, the samples were analyzed using MALDI-TOF. For further identification, we performed the sequencing of specific gene regions, namely ITS1 (Internal Transcribed Spacer), 5.8S, ITS2 rDNA gene cluster regions, and the D1/D2 domain of the LSU (Large Subunit) gene. Genomic DNA extraction followed the method described by Cardinali and coworkers [18]. The amplification of target gene regions was carried out using FIREPole^®^ Taq DNA Polymerase from Solis BioDyne, Tartu, Estonia, with the ITS1-NL4 primer pair (ITS1: 5′-TCCGTAGGTGAACCTGCGG, NL4: GGTCCGTGTTTCAAGACGG). The amplification protocol consisted of an initial denaturation at 94 °C for 3 min, followed by 30 amplification cycles (94 °C for 1 min, 54 °C for 1 min, and 72 °C for 1 min), and a final extension at 72 °C for 5 min. The amplicons were sequenced in both directions using ABI PRISM technology by MACROGEN, Milan, Italy with the same primers used for amplification. The resulting reads of each strain, contained in FASTQ files, were analyzed using Biolomics [19]. The Sanger sequences of the two strains are stored in GenBank with the accession numbers PP919275 (Strain 1) and PP919275 (Strain 2).

### 2.2. Biofilm-Forming Ability

To assess the biofilm-forming ability of the strains, we cultured them in YPD medium (yeast extract 1%, peptone 1%, dextrose 1%) at 37 °C for 24 h at 120 rpm. The cellular suspension was calibrated in a modified RPMI medium [20] to achieve an OD600 value of 0.1. Then, 100 µL of the standardized cell suspensions were seeded into selected wells of a 96-well microtiter plate. Three biological replicates for each strain were performed, with each one in triplicate. The plate was incubated for 2 h at 37 °C to prime the biofilm formation. After removing the inoculum from each well, a multichannel pipette was used to wash each well three times with PBS. Subsequently, 100 µL of the fresh RPMI medium was added to each well. Following 24 h of growth at 37 °C, the plate was collected, and the biofilm was subjected to three different measurements for each strain. The first measurement was taken at 405 nm, which involved assessing the washed biofilm. The second measurement was taken at 570 nm after performing a crystal violet staining procedure. Lastly, the third measurement was taken at 492 nm after conducting the XTT Reduction Menadione Assay (XRMA) using the TECAN Infinite F200 plate reader (Tecan Trading AG, located in Mannedorf, Switzerland).

### 2.3. Determination of Growth Velocity

The strains were cultured in liquid YPD medium at 25 °C for 24 h under constant circular shaking (120 rpm). The cellular suspension was calibrated to achieve OD_600_ = 0.1 and, subsequently, aliquoted in a 96-multiwell plate. The cellular suspension was grown in the Tecan infinite plate reader for 24 h, under continuous shaking conditions at three different growth temperatures, 25 °C, 37 °C and 39 °C, in the same medium (YPD), and six replicates for each strain at each growth condition were produced. The optical density was collected every 5 min, and the growth curves were collected using Magellan v 7.5 software. The data were analyzed using MS Excel.

Growth rates were calculated as the reciprocal of the generation time according to the following Formula (1), where GR is the growth rate (in h^−1^), and T_i_ and T_f_ (in h) are, respectively, the initial and final time point considered within the exponential phase, and D_i_ and D_f_ (in OD_405_) are the cell densities relative to the two time points. 

Formula (1) is given as follows:

(1) $$GR=\frac{\log_{2}\left(\frac{D_{f}}{D_{i}}\right)}{T_{f}-T_{i}}$$

## 3. Results

### 3.1. Case Reports

#### 3.1.1. Case 1

A 57-year-old male patient with a history of non-alcoholic fatty liver disease (NAFLD)-related cirrhosis underwent liver transplantation. After eight months post-transplantation, he experienced acute T-cell-mediated graft rejection, which was successfully managed with high-dose corticosteroids. Subsequently, the patient developed biliary stenosis due to a size discrepancy at the bile duct anastomosis site. Endoscopic retrograde cholangiopancreatography (ERCP) was performed, during which choledocholithiasis was identified and managed by plastic biliary stent placement. Unfortunately, the ERCP procedure resulted in acute pancreatitis, necessitating intensive care unit (ICU) admission. During his ICU stay, the patient developed a fever and impaired consciousness. A rectal swab revealed the presence of *Klebsiella pneumoniae* carbapenemase (KPC)-producing *Klebsiella pneumoniae*. An abdominal CT scan demonstrated acute peripancreatic fluid collections and meropenem and tigecycline combination anti-infective therapy was started. Percutaneous transhepatic cholangiography confirmed intrahepatic and extrahepatic biliary obstruction and an internal-external percutaneous biliary drainage was placed. However, two days later, the patient developed septic syndrome, and blood cultures revealed the presence of *C. lusitaniae* (Strain 1, or CMC 2177) and KPC-producing *K. pneumoniae*. Tigecycline was stopped, and ceftazidime–avibactam, fosfomycin, meropenem, and caspofungin were initiated. Notwithstanding antifungal treatment with caspofungin, the patient experienced persistent *C. lusitaniae* candidemia. 1,3-β-D-glucan (BDG) testing remained consistently negative. Transthoracic (TTE) and transesophageal (TEE) echocardiography ruled out endocardial vegetations, and fundus examination (FE) excluded endophthalmitis. To address the persistent candidemia, the antifungal agent was switched from caspofungin to fluconazole after Antimicrobial Susceptibility Testing, which revealed a fluconazole sensitivity. Unfortunately, the patient’s condition deteriorated, and he developed septic shock, ultimately leading to his demise despite aggressive antimicrobial and antifungal therapy.

#### 3.1.2. Case 2

A 40-year-old male patient with a history of high blood pressure and asthma had severe COVID-19 two months before admission to our hospital. He received treatment with methylprednisolone and tocilizumab during hospitalization in Belgrade. However, he subsequently developed fulminant hepatitis B and acute liver failure, characterized by significantly elevated liver enzymes, a positive hepatitis B surface antigen (HBsAg), positive hepatitis B core antibody (anti-HBc IgM), and high viral load (HBV DNA). Due to the severity of his liver failure, he was transferred to Udine for intensive care unit (ICU) admission and liver transplantation. Immunosuppression induction with basiliximab and methylprednisolone was initiated, along with a delayed administration of tacrolimus. Microbiological surveillance samples revealed respiratory tract colonization by *Acinetobacter baumannii* and methicillin-sensitive *Staphylococcus aureus* (MSSA). A rectal swab and a urine culture indicated the growth of NDM-producing *Klebsiella pneumoniae* (NDM: New Delhi metallo-ß-lactamase). On post-transplant day 2, the patient developed a fever and hemodynamic instability. Signs of surgical wound infection with purulent discharge were observed. Antimicrobial therapy with cefiderocol, fosfomycin, and anidulafungin was initiated. On post-transplant day 12, the patient underwent relaparotomy for surgical site hematoma, and blood cultures and ascitic fluid cultures were consistently identified as NDM-producing *K. pneumoniae*; histologic examinations of a liver biopsy revealed acute T-cell mediated rejection. High-dose corticosteroids were initiated. On post-transplant day 37, angioplasty and stenting were performed to address hepatic artery stenosis, but the restoration of arterial liver perfusion was unsuccessful. Antimicrobial therapy was withdrawn on post-transplant day 50, after 28 days, since the first negative follow-up blood culture. Four days later, the patient experienced an episode of septic shock, requiring fluid resuscitation and vasopressors. Blood cultures and ascitic fluid cultures are still identified as NDM-producing *K. pneumoniae*. Blood cultures were also positive for *C. lusitaniae* (Strain 2, or CMC 2232). A combination antimicrobial therapy with Aztreonam, ceftazidime-avibactam, tigecycline, and anidulafungin was initiated. On post-transplant day 72, because of the impending life-threatening toxic liver syndrome, two-stage liver re-transplantation (re-OLTx) was performed. Throughout his post-re-OLTx course, the patient faced a complicated intra-abdominal infection with a fluid collection that consistently grew *C. lusitaniae* and persistent secondary candidemia due to *C. lusitaniae* despite echinocandin antifungal therapy. TTE and TEE ruled out endocardial vegetations, and FE excluded endophthalmitis. Of note, BDG testing remained negative throughout the entire disease course. The addition of liposomal amphotericin B (L-AmB) to antifungal therapy successfully cleared the *C. lusitaniae* candidemia. 

### 3.2. Identification and Typing

**Figure 1:**
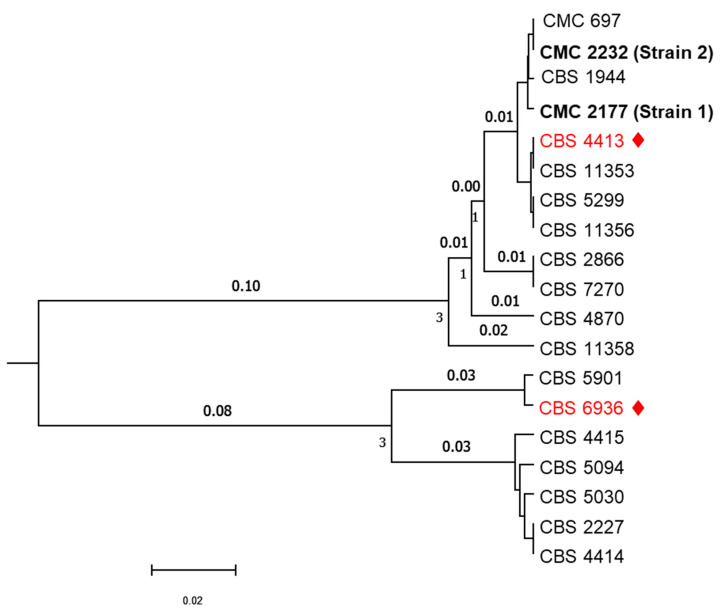
UPGMA phylogenetic tree of *C. lusitaniae* strains, type strains are indicated in red (red text and red square). Strains 1 and 2 are indicated with bold text.

The sequencing of the marker sequence ITS1-ITS2 and LSU confirmed the identity of the fungal species as *C. lusitaniae*. This yeast species is known to be very variable in terms of LSU [15], as demonstrated by the presence of two major clades in the UPGMA tree obtained by the analysis of the concatenated sequences (Figure 1). 

This UPGMA phylogenetic tree illustrates the evolutionary relationships among *C. lusitaniae* strains. The tree is constructed based on concatenated ITS (Internal Transcribed Spacer) and LSU (Large Subunit) marker sequences. The two type strains of *C. lusitaniae*, namely CBS 4413 and CBS 6936, are highlighted in red, and Strain 1 (CMC 2177) isolated from Case 1 and Strain 2 (CMC 2232) isolated from Case 2 are also included. Fifteen *C. lusitaniae* strains retrieved from Mycobank contribute to the overall diversity depicted in the tree.

Interestingly, the two isolated strains are very close to CBS 4413, formerly the type-strain of *Candida lusitaniae*, and are placed in the upper clade, populated mostly by strains found in specimens of warm-blooded animals. Strain 2 appears identical to strain CMC 697, isolated from food in Lebanon, and very similar to Strain 1. 

### 3.3. Antifungal Resistance and Growth Curves

Using the Vitek^®^ 2 AST, the fluconazole MIC for both Strains 1 and 2 was determined to be 0.5 mg/dL. The E-test method was used only for strain 1, yielding the following MIC values: anidulafungin 0.0312 mg/L, amphotericin B 0.25 mg/L, 5-fluocytosine 0.125 mg/L, and micafungin 0.0312 mg/L The susceptibility of the two strains to liposomal amphotericin B, isavuconazole and fluconazole was tested following the broth microdilution method for antifungal susceptibility testing (AFST), obtaining the MIC values reported in Table 1. 

**Figure 2:**
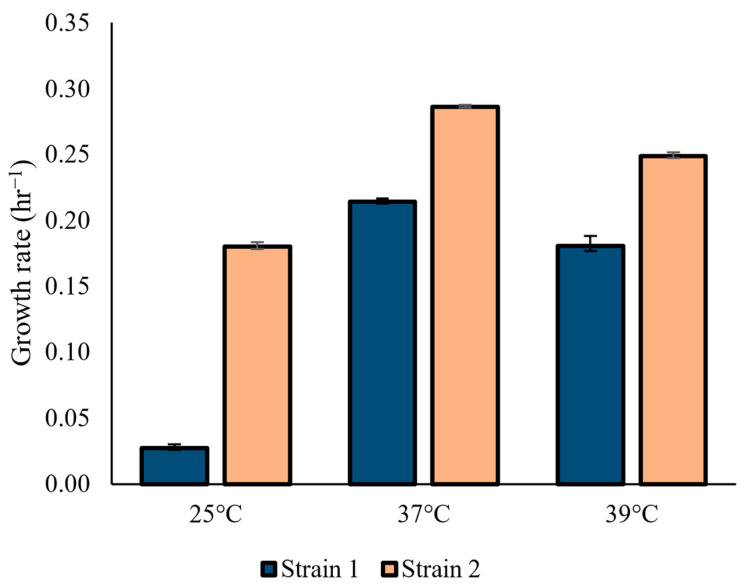
Growth rates of Strain 1 and Strain 2 at 25 °C, 37 °C, and 39 °C.

For further discrimination, the two strains were investigated by evaluating the growth velocity at different temperatures, 25 °C, 37 °C, and 39 °C, which were chosen in order to mimic room and body temperature as well as a temperature that could be reached in fever-like conditions. Both strains showed a similar growth rate at 37 °C and 39 °C, with Strain 1 growing faster than Strain 2 at both temperatures (Figure 2).

The strains isolated from Case 1 and Case 2 (Strain 1 and 2, respectively) were cultured in YPD at temperatures of 25 °C, 37 °C, and 39 °C. Their growth was monitored over 24 h. Exponential phases, retrieved from growth curves, were used to calculate the growth rate of the two strains. Standard deviation WAS reported for each strain grown at each temperature.

The most notable difference between the two isolates was observed at 25 °C. While Strain 1 exhibited minimal growth with a growth rate of 0.027 h^−1^, Strain 2 displayed a significantly higher growth rate of 0.18 h^−1^. For both Strain 1 and Strain 2, the optimal temperature of growth was 37 °C, 0.21 h^−1^, and 0.28 h^−1^, respectively. Growth at 39 °C showed a decrease in the growth rate to 0.18 h^−1^ for Strain 1 and to 0.24 h^−1^ for Strain 2.

Finally, both strains were tested for biofilm formation ability, as described in the Methods section, but no biofilm adhesion or development could be observed even after five days of incubation. 

## 4. Discussion

We report here two cases of invasive fungal infection due to *C. lusitaniae* in immunocompromised patients. *C. lusitaniae* is an uncommon cause of candidemia in patients with solid or hematologic neoplasms [21] and patients undergoing prolonged antibiotic treatments [22], and it can occur as breakthrough fungemia in patients receiving antifungal treatment or antifungal prophylaxis [21]. *C. lusitaniae* is considered intrinsically resistant to AmB, and it has been reported to acquire multidrug resistance in patients exposed to antifungal drugs [6,23,24].

Strain 1 and Strain 2 exhibited AmB MICs of 0.125 mg/L and 0.06 mg/L, respectively. No clinical breakpoints for AmB exist for *C. lusitaniae* [25]. However, a tentative epidemiological cut-off (ECOFF) of 0.5 mg/L for AmB has been released by the EUCAST. The role of ECOFFs is that of distinguishing between a wild-type isolate (without a mechanism of resistance) and a non-wild-type isolate (with an acquired mechanism of drug resistance) [26,27], and they do not classify isolates as susceptible, resistant, or anticipated therapeutic responses. The ECOFF value is the highest MIC value for organisms devoid of phenotypically detectable acquired mechanisms of antifungal drug resistance and corresponds to the MIC where the wild-type distribution of microorganisms ends [28]. Indeed, taking into account the AmB MIC of Strain 1 and Strain 2, both of them might be considered wild-type organisms virtually not harboring acquired mechanisms of phenotypic resistance to AmB. However, it is noted that the two *C. lusitaniae* strains are distinct from each other in various aspects. Firstly, by comparing the MICs of isavuconazole and fluconazole, it is evident that these are much higher for Strain 1 (>32 mg/L and >256 mg/L, respectively) than for Strain 2 (0.25–0.5 mg/L and 2–4 mg/L, respectively). MIC-independent non-species-related *Candida* fluconazole breakpoints, mainly determined based on PK/PD studies, were 2 mg/L for susceptibility and 4 mg/L for resistance [29]. Therefore, we could reasonably assume that Strain 2, unlike Strain 1, is “susceptible, standard dosing regimen” or “susceptible, increased exposure” to fluconazole, and in any case, not overtly resistant. It is currently well known that azole antifungal resistance in *Candida* is mainly related to the following four fundamental mechanisms: (1) the upregulation of genes encoding multidrug efflux transporters, including ATP-binding cassette (ABC) transporters, as well as the major facilitator superfamily (MFS) efflux pumps (with the formers accepting a broad range of azole antifungals and the latter exclusively recognizing fluconazole); (2) increased expression of the gene ERG11, which encodes lanosterol 14-α-demethylase, the enzyme that is targeted by azole antifungals; (3) amino acid substitution in Erg11p, thus reducing the affinity of azoles for their target; and (4) changes in sterol composition exemplified by those changed due to the altered activity of sterol Δ^5,6^ desaturase encoded by the Erg3 gene [30,31]. Modifications in the ergosterol synthetic pathway due to mutation within Erg2 or Erg3 genes or a gene governing the expression of Erg3 might be associated with cell wall alterations, phenotypic switching, and co-resistance to AmB due to the reduced ability of AmB to access the fungal membrane and to build ion channels [10,22,32,33,34,35]. Given that azole resistance in Strain 1 is not correlated with resistance to AmB, we could deduce that, in this scenario, the microorganism exhibits either an overactivity of efflux pumps or a modification of the intracellular target of azoles rather than an alteration in the ergosterol biosynthesis pathway. We speculated that a non-target-site-related mechanism might underlie azole resistance in Strain 1, as already reported by Asner and colleagues [23]. In addition, Strain 1 demonstrated a slower growth rate compared to Strain 2. Strain 2 demonstrates faster growth and higher susceptibility to drugs compared to Strain 1 and might be more likely associated with the environment or food-borne sources, while Strain 1 is presumed to have been acquired in a hospital setting. In fungi, the nonheritable tolerance to antifungal agents was associated with slower growth in the presence of drugs [36]. In the present case, Strain 1 showed higher tolerance to the antimycotics and slower growth in a drug-free medium. Further studies are necessary to evaluate whether this behavior may be ascribed to some form of tolerance or if it is a mere coincidence. It is noteworthy that the development of drug resistance is associated with a trade-off of fitness costs in *Candida* spp. [37,38]. The overexpression of the efflux pump, diminished growth at low temperatures, and levels of growth at higher temperatures, comparable to that of wild-type strains, have already been observed in molds [39], and this could have been the case in our strains as well. Interestingly, both strains exhibited minimal or no production of beta-D-glucan, a cell wall component commonly associated with invasive fungal infections. This finding suggests that other mechanisms may be responsible for their pathogenicity, such as cell wall integrity pattern alteration [40], that might be associated with echinocandin resistance [41]. Both strains were found to be susceptible to L-AmB, and in case 2, *C. lusitaniae* infection was successfully cleared less than 10 days after initiating L-AmB treatment. These findings suggest that L-AmB could be an effective treatment option for *C. lusitaniae* indeed [8].

## 5. Conclusions

The two strains studied belong to a species with extended LSU variability, which is present in a number of environments, although the clinical isolates are more frequent in culture collections and databases. In spite of the taxonomic similarity and the proximity of the time and place of isolation, the two strains exhibited significant differences at the physiological level and in their resistance to antimycotics. Interestingly, the least antifungal-resistant strain (Strain 2) appeared very close to a food isolate and exhibited faster growth at 25 °C in comparison to Strain 1, suggesting that it could have originated out of the clinical environment in ecological niches with intermediate temperatures. On the other hand, Strain 1 was more resistant to antifungal agents and displayed better growth at 37 °C than at 25 °C, suggesting an origin in clinical settings with a selection for faster growth at the human body temperature than at room temperature. It is also tempting to correlate the slower growth with the higher resistance, postulating that the former gives more time to the extrusion mechanisms to remove antifungal drugs, but the data available is not sufficient to sustain such a hypothesis, and further work is necessary in this direction. Finally, the presence of strains derived from food, such as the CBS 6936 and CMC 697, and from the outer environment in close relationship with clinical isolates raises the question of the origin of the pathogenic strains and the selection mechanisms to make them more aggressive in hospital conditions. 

## Figures and Tables

**Table 1:** Broth microdilution antifungal susceptibility testing (AFST) of Strains 1 and 2 for isovuconazole, fluconazole, and liposomal amphotericin B.

	Isovuconazole	Fluconazole	L-AMB
	(mg L^−1^)		
Strain 1	>32	>256	0.125
Strain 2	0.25–0.5	2–4	0.06

## Data Availability

Data are available upon request.
